# Tumor metabolic plasticity in therapy resistance: from the Warburg effect to mitochondrial hijacking

**DOI:** 10.7150/thno.131708

**Published:** 2026-02-26

**Authors:** Yen-Dun Tony Tzeng, Emmanuel Naveen Raj, Shih-Hsuan Cheng, Su-Boon Yong, Shih-Chieh Lin, Ren-Wang Peng, Chia-Jung Li

**Affiliations:** 1 Department of Surgery, Kaohsiung Veterans General Hospital, Kaohsiung 813, Taiwan.; 2 Department of Obstetrics and Gynecology, Kaohsiung Veterans General Hospital, Kaohsiung 813, Taiwan.; 3 Department of Allergy and Immunology, China Medical University Children's Hospital, Taichung 404, Taiwan.; 4 Research Center for Allergy, Immunology, and Microbiome (A.I.M.), China Medical University Hospital, Taichung 404, Taiwan.; 5 Department of Medicine, College of Medicine, China Medical University, Taichung 404, Taiwan.; 6 Institute of Basic Medical Sciences, College of Medicine, National Cheng Kung University, Tainan 701, Taiwan.; 7 Department of General Thoracic Surgery, Inselspital, Bern University Hospital, Bern 3010, Switzerland.; 8 Department of BioMedical Research (DBMR), University of Bern, Bern 3010, Switzerland.; 9 Institute of BioPharmaceutical Sciences, National Sun Yat-sen University, Kaohsiung 804, Taiwan.; 10 National Museum of Marine Biology & Aquarium, Pingtung 944, Taiwan.; 11 Center of General Education, Cheng Shiu University, Kaohsiung 833, Taiwan.

**Keywords:** tumor metabolism, drug-tolerant persisters (DTPs), reverse Warburg effect, mitochondrial hijacking, immunometabolism, oxidative phosphorylation

## Abstract

The clinical efficacy of targeted cancer therapies is persistently undermined by the emergence of acquired resistance. While secondary genetic mutations are well-characterized, increasing evidence implicates non-genetic metabolic reprogramming as a primary driver of survival during the initial phase of treatment. This review elucidates the concept of "Metabolic Shapeshifters"—specifically, drug-tolerant persister cells (DTPs) that dynamically adapt their bioenergetic machinery to evade therapeutic stress. We examine the plasticity between the classical Warburg Effect and the Reverse Warburg Effect, describing how DTPs shift from a glucose-addicted proliferative state to a quiescent phenotype strictly reliant on mitochondrial oxidative phosphorylation (OXPHOS) and fatty acid oxidation. Crucially, we highlight a paradigm shift from intracellular reprogramming to intercellular "organelle parasitism." Recent breakthroughs demonstrate that DTPs actively hijack functional mitochondria from infiltrating immune cells and the stromal network via tunneling nanotubes (TNTs). This predatory behavior not only restores the tumor's respiratory capacity but also induces metabolic exhaustion in T cells, thereby orchestrating immune evasion. Finally, we delineate emerging therapeutic strategies designed to dismantle this metabolic fortress. By targeting the "Achilles' heel" of mitochondrial dependency, disrupting the physical infrastructure of organelle hijacking, and revitalizing immunometabolism, we propose a multi-pronged framework to eradicate DTPs and prevent clinical relapse.

## 1. Introduction

The conceptual framework of cancer metabolism has undergone a paradigm shift over the past century. Initially dominated by the observation of Otto Warburg in the 1920s, the field long operated under the assumption that malignant cells are characterized by a static reliance on aerobic glycolysis due to defective mitochondria, a phenomenon known as the "Warburg Effect" 1. This metabolic reprogramming is characterized by elevated aerobic glycolysis and increased lactate secretion, reflecting a redistribution of glucose-derived carbon flux rather than a simultaneous enhancement of lactate production and anabolic biosynthesis from the same carbon pool. When the majority of glucose-derived carbon is converted to lactate, its availability for branching biosynthetic pathways such as the pentose phosphate pathway or serine biosynthesis is necessarily reduced, underscoring an inherent trade-off in metabolic flux allocation 2, 3.

However, the dogmatic view that cancer cells are strictly glycolytic and possess dysfunctional mitochondria has been fundamentally challenged by accumulating evidence. These studies demonstrate that most tumors retain functional mitochondria and exhibit significant metabolic heterogeneity 4. Recent advances in metabolomics and single-cell sequencing have revealed that tumor metabolism is not a fixed phenotype but a highly plastic state that evolves in response to microenvironmental cues and therapeutic stress 5. This "Metabolic Plasticity" enables cancer cells to switch between glycolysis and oxidative phosphorylation (OXPHOS), a flexibility that is particularly evident in the context of the tumor microenvironment (TME) 6. A critical manifestation of this plasticity is the "Reverse Warburg Effect," which describes a metabolic symbiosis wherein hypoxic or oxidatively stressed cancer cells coerce neighboring cancer-associated fibroblasts (CAFs) to undergo aerobic glycolysis. These CAFs secrete energy-rich fuels, such as lactate, pyruvate, and ketone bodies. Subsequently, these metabolites are taken up by oxidative tumor cells to fuel mitochondrial respiration and ATP production 7, 8.

This metabolic adaptability poses a formidable challenge to current targeted therapies and immunotherapies. While kinase inhibitors, such as EGFR or BRAF inhibitors, effectively eliminate rapidly proliferating and glycolytic tumor cells, they frequently fail to eradicate a subpopulation known as DTPs 9, 10. Emerging research indicates that DTPs exploit metabolic plasticity to survive lethal drug exposure. They often achieve this by shifting their dependency from glycolysis to mitochondrial OXPHOS and lipid metabolism, thereby entering a quiescent and diapause-like state 11. Consequently, targeting the metabolic shapeshifting capability of these cells, specifically the dynamic transition between Warburg and Reverse Warburg phenotypes, represents a novel and urgent therapeutic frontier 12. This review provides a comprehensive analysis of the molecular mechanisms governing metabolic plasticity in the context of therapy resistance. We dissect the signaling pathways regulating the switch between glycolytic and oxidative states, examine the role of the Reverse Warburg Effect in sustaining DTPs, and evaluate emerging therapeutic strategies designed to dismantle this metabolic flexibility to overcome drug resistance in cancer. Unlike prior reviews that primarily catalog metabolic pathways or targets, this review reframes tumor metabolism through the lens of therapy resistance and relapse, emphasizing transient drug-tolerant persister states, intercellular metabolic coupling, and multi-layer intervention strategies.

## 2. The Metabolic Spectrum: Warburg, Reverse Warburg, and the Hybrid State

**Figure 1:**
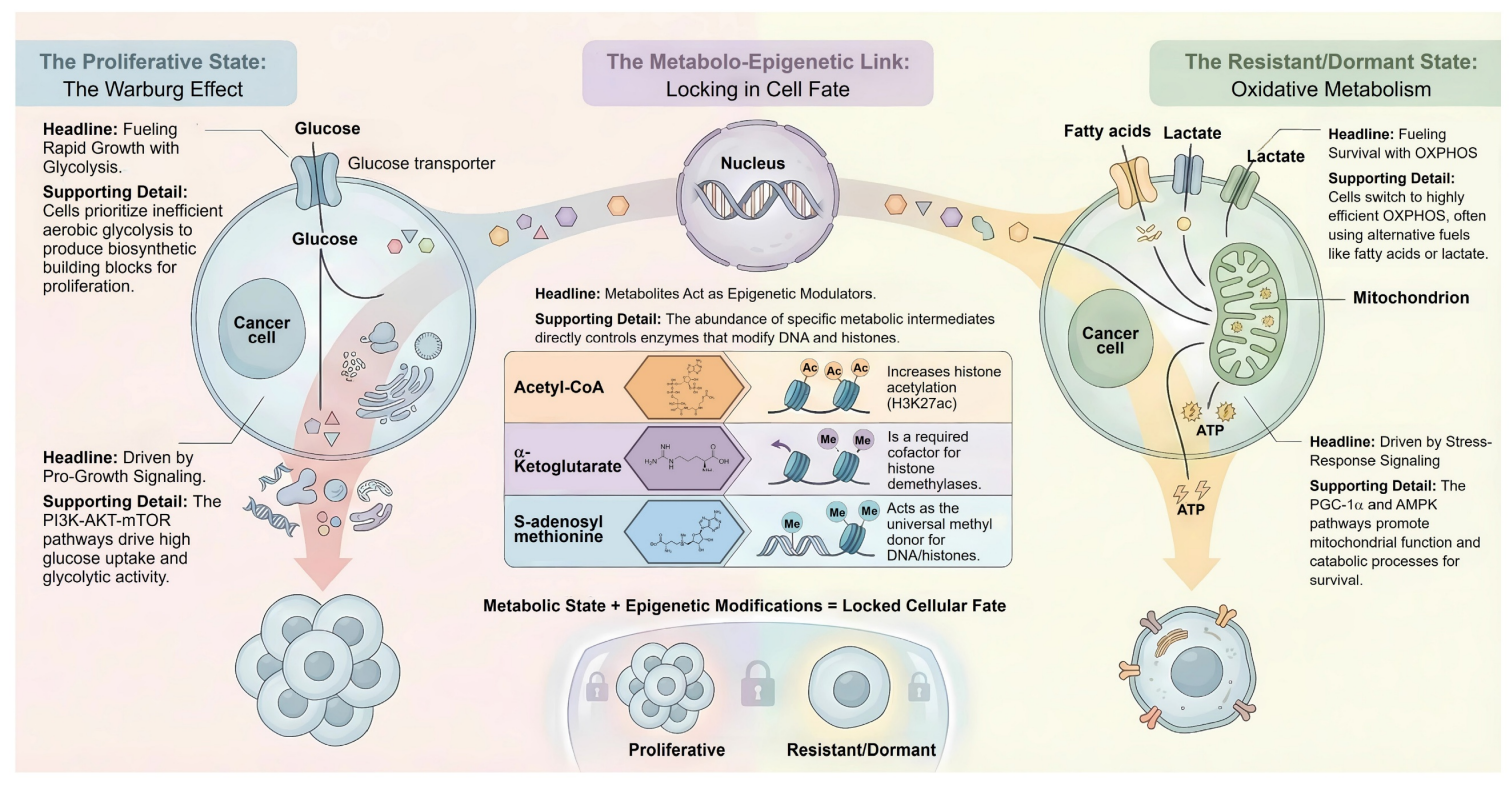
** The spectrum of metabolic plasticity and the metabolo-epigenetic axis in cancer.** The figure illustrates the dynamic transition between two distinct cell fates driven by metabolic reprogramming. The Proliferative State: Characterized by the Warburg Effect, where oncogenic signaling (c-MYC, PI3K-AKT-mTOR) drives high glucose uptake and aerobic glycolysis to fuel rapid biosynthesis. The Resistant/Dormant State: Represents the phenotype of DTPs, which shift towards mitochondrial OXPHOS and fatty acid oxidation (FAO). This survival mode is orchestrated by stress-response signaling (PGC-1α, AMPK). The Metabolo-Epigenetic Link: Depicts how metabolic intermediates serve as substrates for chromatin-modifying enzymes to "lock" cell fate. High levels of Acetyl-CoA drive histone acetylation (H3K27ac) to maintain stemness, while α-KG and S-adenosylmethionine regulate DNA and histone methylation, thereby translating metabolic flux into stable epigenetic landscapes.

Tumor metabolism is traditionally viewed as a binary choice between glycolysis and oxidative phosphorylation. However, contemporary evidence suggests that cancer cells exist along a continuous metabolic spectrum. This plasticity allows malignant cells to dynamically adapt their metabolic machinery to meet the bioenergetic and biosynthetic demands of a hostile microenvironment 5 (**Figure 1**).

### 2.1 The Warburg Effect Revisited: Anabolic Requirements over ATP Efficiency

Although aerobic glycolysis, also known as the Warburg Effect, is inefficient in terms of ATP generation relative to oxidative phosphorylation, it confers a significant survival advantage to rapidly proliferating cells 13. The Warburg effect should not be interpreted as a unidirectional mechanism that simultaneously maximizes lactate production and biosynthetic flux. Rather, it represents a metabolic state that favors rapid ATP generation, maintenance of redox balance, and metabolic flexibility, while constraining glucose-derived carbon entry into anabolic pathways when lactate secretion predominates 2, 3. These include the pentose phosphate pathway for nucleotide synthesis and the hexosamine pathway for protein glycosylation 14, 15. Key signaling nodes driving this phenotype include the PI3K-AKT-mTOR axis 16. This pathway upregulates the surface expression of glucose transporter 1 (GLUT1) and enhances the activity of key glycolytic enzymes such as Hexokinase 2 and Pyruvate Kinase M2 17. While this phenotype dominates in the early stages of tumorigenesis, recent *in vivo* isotope tracing studies have revealed that tumors in their native environment often retain significant oxidative capacity. Crucially, strictly glycolytic cells often lack the metabolic flexibility required to survive the fluctuating nutrient availability typical of the metastatic cascade or the drug-induced stress environment 18.

### 2.2 The Reverse Warburg Effect: Stromal-Epithelial Metabolic Symbiosis

In contrast to the cell-autonomous nature of the Warburg Effect, the "Reverse Warburg Effect" describes a parasitic metabolic relationship between tumor cells and the surrounding stromal compartment, particularly cancer-associated fibroblasts. This paradigm suggests that oxidative stress in cancer cells acts as a trigger to remodel the stroma 19. Cancer cells induce oxidative stress in neighboring fibroblasts by secreting reactive oxygen species and exosomes carrying metabolic modulators 20. This signaling triggers the degradation of stromal Caveolin-1 via autophagy-lysosomal pathways 21. The loss of Caveolin-1 stabilizes Hypoxia-inducible factor 1-alpha in the aerobic fibroblasts, which forces them to undergo aerobic glycolysis and effectively mimics the Warburg Effect 22. Consequently, these metabolically reprogrammed fibroblasts produce high levels of energy-rich metabolites, specifically L-lactate, pyruvate, and ketone bodies 23. These fuels are exported into the tumor microenvironment via monocarboxylate transporter 4 24. Adjacent cancer cells act as metabolic parasites to uptake these fuels via monocarboxylate transporter 1 25. Inside the cancer cell, lactate is converted back to pyruvate by lactate dehydrogenase B and funneled directly into the mitochondrial TCA cycle for high-efficiency ATP production via oxidative phosphorylation 8. This "Two-Compartment Tumor Metabolism" model explains why many tumors with functional mitochondria are resistant to anti-glycolytic therapies, as they do not rely on glucose for fuel but rather utilize stromal-derived lactate to power their mitochondria 7.

### 2.3 Molecular Switches of Metabolic Plasticity

The transition between glycolytic and oxidative phenotypes is strictly governed by a complex network of transcription factors and nutrient sensors. Central to this regulation is the interplay between HIF1-α and c-MYC 26. While HIF1-α acts as the master regulator of the glycolytic program by upregulating GLUT1 and LDH-A in response to hypoxia, c-MYC drives a distinct and context-dependent transcriptional program 27. Although c-MYC promotes glycolysis in proliferating cells, it plays a divergent role in drug-resistant contexts where it frequently cooperates with the co-activator PGC-1α to enhance mitochondrial mass and respiratory capacity 28. This c-MYC and PGC-1α axis enables tumor cells to maintain bioenergetic stability even when glycolytic pathways are suppressed by targeted therapies 11. Beyond transcriptional control, the AMP-activated protein kinase serves as the critical energy gauge of the cell and orchestrates metabolic shifts in real-time 29. Under conditions of metabolic stress characterized by a low ATP to ADP ratio, AMP-activated protein kinase activation suppresses mTORC1 signaling and thereby inhibits anabolic processes such as protein synthesis 30. Concurrently, it promotes catabolic survival processes, including fatty acid oxidation and mitophagy. This metabolic rewiring pushes the cancer cell towards a quiescent, oxidative, and drug-tolerant state, effectively shielding it from cytotoxic therapies that target rapidly dividing cells 31. Furthermore, this oxidative shift is reinforced by the SIRT1-PGC-1α axis 32. The NAD-dependent deacetylase SIRT1 functions as a sensor of nutrient deprivation and activates PGC-1α via deacetylation 33. This activation is frequently observed in drug-resistant tumors, allowing them to maximize ATP production from alternative fuels when glucose transport is compromised 34.

### 2.4 The Metabolo-Epigenetic Axis: Locking in the Plasticity

While signaling pathways orchestrate rapid metabolic responses, the persistence of the drug-tolerant phenotype requires a durable cellular memory established through metabolo-epigenetics 35. The transition to a mitochondrial-dependent state in persister cells fundamentally alters the intracellular pool of key substrates required for chromatin-modifying enzymes 36, 37. Specifically, acetyl-CoA serves as the obligatory acetyl donor for histone acetyltransferases. In persister cells, the shift towards fatty acid oxidation and the upregulation of ATP-citrate lyase maintain a high nuclear pool of acetyl-CoA 38. This drives the hyperacetylation of histones, such as H3K27ac, at the promoters of genes governing stemness and antioxidant defense 39, 40. This epigenetic opening allows persister cells to maintain a dedifferentiated state refractory to targeted therapies. Conversely, the TCA cycle intermediate α-Ketoglutarate acts as a critical cofactor for TET DNA demethylases and JmjC domain-containing histone demethylases 41. However, in the context of the reverse Warburg effect or hypoxia, the accumulation of oncometabolites such as 2-Hydroxyglutarate or Succinate acts as competitive inhibitors of these enzymes 42. The resulting inhibition leads to a global hypermethylation phenotype, which silences differentiation genes and tumor suppressor loci 43. Epigenetic regulation in cancer cells is influenced by one-carbon metabolism, primarily through serine-derived one-carbon units that support nucleotide synthesis and methyl donor availability. However, the extent to which one-carbon metabolism directly governs epigenetic plasticity, particularly in therapy-tolerant or persister states, remains context dependent and incompletely defined 44, 45. S-adenosylmethionine, derived from methionine and ATP, serves as the universal methyl donor for DNA and histone methyltransferases. Alterations in folate-dependent one-carbon metabolism have been proposed to support methyl donor homeostasis under metabolic stress; however, direct experimental evidence demonstrating sustained maintenance of intracellular S-adenosylmethionine levels in cancer persister cells remains limited, and this relationship has largely been inferred from indirect metabolic or epigenetic readouts 46. Methionine availability has been implicated in supporting cancer cell growth, survival, and epigenetic regulation, and LAT1-mediated amino acid transport is upregulated in multiple tumor types. Nevertheless, direct evidence demonstrating a specific dependency of persister cells in solid tumors on LAT1-mediated methionine uptake remains incomplete, and existing studies primarily address bulk tumor cell populations or non-cancer cell types 47-49. High intracellular S-adenosylmethionine levels drive the trimethylation of Histone H3 at Lysine 4 at the promoters of drug-tolerance genes 50, 51. Modulation of methionine availability can influence intracellular S-adenosylmethionine levels and histone methylation states; however, the directionality and functional consequences of these epigenetic changes are highly context dependent. Notably, methionine metabolism has been shown to enhance histone methylation and immune cell function in specific settings, underscoring that the effects of methionine restriction or supplementation cannot be generalized across tumor and immune compartments or across distinct therapeutic contexts 52. Thus, metabolic reprogramming in DTPs acts not merely as a survival mechanism but as the upstream signal that rewrites the epigenetic landscape, ensuring that transient drug tolerance evolves into stable resistance (**Figure 1**).

## 3. Metabolic Plasticity as a Driver of Kinase Inhibitor Resistance

The clinical efficacy of targeted therapies, particularly tyrosine kinase inhibitors, is often limited by the inevitable emergence of acquired resistance. While secondary genetic mutations such as EGFR T790M are well-documented, increasing evidence points to metabolic reprogramming as a rapid and adaptive mechanism enabling survival during the initial treatment phase 53. Oncogenic signaling pathways, including EGFR, RAS, and BRAF, primarily drive the Warburg Effect. Consequently, acute kinase inhibition imposes a sudden energy crisis. Cells that survive acute therapeutic stress, termed drug-tolerant persisters, undergo broad and durable metabolic rewiring that distinguishes them from the pre-treatment population, rather than simply reverting to their original metabolic programs 55. They function as metabolic shapeshifters that transition from a glycolytic phenotype to a state reliant on mitochondrial oxidative phosphorylation and fatty acid oxidation 56. This switch allows residual cells to maintain bioenergetics independent of the original oncogenic driver.

### 3.1 The Shift to Mitochondrial Dependency

In models of EGFR-mutant lung cancer and BRAF-mutant melanoma, therapy resistance is characterized not merely by increased mitochondrial biogenesis, but by a functional reliance on oxidative metabolism that persists under therapeutic stress 57. *In vivo* isotope tracing studies have confirmed that tumors in their native microenvironment retain significant oxidative capacity and often utilize non-glucose fuels to power the TCA cycle 7. Following tyrosine kinase inhibitor treatment, surviving DTPs exhibit a structural and functional reorganization of the electron transport chain, resulting in an elevated reserve respiratory capacity 58, 59. Crucially, this mitochondrial dependency is validated *in vivo*, where DTPs effectively scavenge stromal-derived lactate and fatty acids to maintain ATP production even when glycolysis is pharmacologically or microenvironmentally restricted 8, 56. The therapeutic relevance of this shift is demonstrated by the potent anti-tumor activity of mitochondrial Complex I inhibitors in preclinical models, which induces metabolic collapse specifically in these mitochondria-addicted subpopulations 60.

### 3.2 Mitochondrial Dynamics: The Morphology of Survival

The concept of the shapeshifter extends beyond metabolic flux to characterize the physical architecture of the mitochondrial network itself 61. Under therapeutic stress, DTPs frequently undergo a structural metamorphosis known as mitochondrial hyperfusion 62. This elongation process is orchestrated by the upregulation of inner and outer membrane fusion proteins, specifically OPA1 and MFN1/2, concomitant with the suppression of the fission regulator DRP1 63. This fused mitochondrial network confers two distinct survival advantages essential for the persister state. First, the interconnected morphology optimizes the electrochemical gradient across the inner membrane, thereby maximizing ATP production efficiency per unit of substrate 64. This is a critical adaptation for cells operating in a nutrient-deprived microenvironment. Second, and perhaps more importantly, hyperfusion acts as a physical barrier against quality control mechanisms. Elongated mitochondria are sterically hindered from being engulfed by autophagosomes, effectively allowing persister cells to evade mitophagy 65. By circumventing this degradation pathway, persister cells preserve their metabolic engines during prolonged drug exposure 66. However, this state is reversible. Upon acquiring secondary mutations or environmental cues that trigger relapse, the mitochondrial network shifts back toward fission to facilitate organelle distribution and rapid cell proliferation 67. This underscores the dynamic nature of these organelles in dictating cell fate.

### 3.3 DTP Cells (DTPs) and Lipid Metabolism

A specific subpopulation of resistant cells, the DTP cells, represents the clinical reservoir of minimal residual disease. These cells enter a quiescent and diapause-like state characterized by suppressed global protein synthesis but highly active lipid metabolism 68, 69. Unlike rapidly dividing cells that synthesize fatty acids for membrane construction, persister cells predominantly rely on Fatty Acid Oxidation to sustain ATP levels and NADPH production 70. This heavy reliance on lipid metabolism comes at a cost as it generates significant levels of lipid peroxides and ROS 71. To counter this oxidative stress, persister cells become exquisitely dependent on the GPX4 pathway to prevent ferroptosis, which is an iron-dependent form of cell death 55. This creates a unique and druggable metabolic vulnerability. While persister cells are resistant to apoptotic agents like chemotherapy or tyrosine kinase inhibitors, their metabolic state renders them hypersensitive to ferroptosis inducers 72, 73. Therefore, the metabolic plasticity that allows these cells to escape initial therapy ironically exposes a new Achilles' heel that can be targeted by inhibiting lipid antioxidant defenses.

### 3.4 The Integrated Stress Response: The Signaling Engine of Persistence

**Figure 2:**
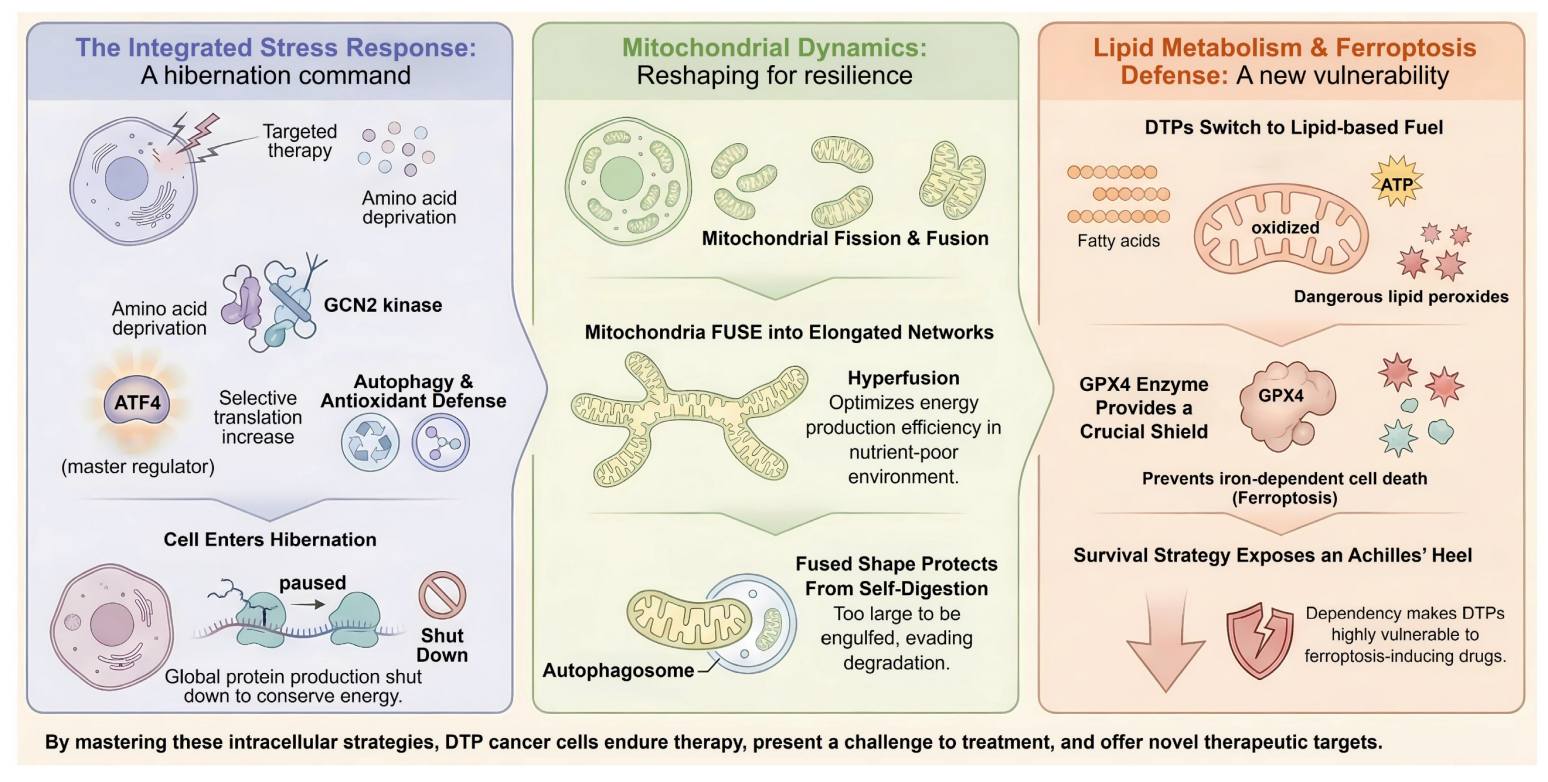
** Signaling networks and intracellular adaptations orchestrating DTP survival.** The figure depicts the three core mechanisms enabling DTP cells to withstand therapeutic stress. The ISR: Therapy-induced stress (amino acid deprivation) activates the kinase GCN2. This triggers a "hibernation" state by shutting down global protein synthesis to conserve energy, while selectively translating the master regulator ATF4 to drive autophagy and antioxidant defense. Mitochondrial Dynamics: DTPs undergo mitochondrial hyperfusion, forming elongated networks. This structural remodeling optimizes OXPHOS efficiency in nutrient-poor conditions and physically prevents organelle degradation by evading mitophagy. Lipid Metabolism and Ferroptosis Defense: DTPs shift their metabolic dependency to FAO for ATP production, which generates toxic lipid peroxides. Survival becomes critically dependent on the enzyme GPX4 to neutralize these peroxides, creating a specific "Achilles' heel" vulnerability to ferroptosis inducers.

Beyond direct metabolic rewiring, the survival of DTPs is orchestrated by a sophisticated signaling network known as the Integrated Stress Response 74. When tumor cells encounter the energy crisis of targeted therapy, the sudden depletion of amino acids and accumulation of reactive oxygen species trigger the phosphorylation of the translation initiation factor eIF2α 75. This event is mediated by four distinct stress-sensing kinases: GCN2 senses amino acid deprivation, PERK senses ER stress, PKR senses dsRNA, and HRI senses heme or mitochondrial stress. The phosphorylation of eIF2α results in a global shutdown of cap-dependent protein synthesis. This effectively puts the cell into hibernation to conserve energy, which is a hallmark of the persister state 76. However, it paradoxically increases the translation of select mRNAs containing upstream open reading frames. The most notable among these is the transcription factor ATF4 77. ATF4 acts as the master regulator of the stress response by inducing genes involved in amino acid biosynthesis such as ASNS and PHGDH, autophagy genes, and antioxidant defense genes like HO-1 78. Recent studies have identified that the GCN2-ATF4 axis is essential for maintaining the pool of biosynthetically active persister cells 79, 80. Crucially, pharmacological inhibition of the integrated stress response (ISR), for example using ISRIB, has been shown to prevent the establishment of the persister state 81, 82. This forces cells to continue dividing under stress and leads to apoptosis. Thus, the ISR functions as the central signal transduction hub that translates metabolic stress into a transcriptional program of persistence 83. Thus, the ISR functions as the central signal transduction hub that translates metabolic stress into a transcriptional program of persistence, coordinating the mitochondrial and lipid adaptations depicted in **Figure 2**.

### 3.5 Autophagy: The Internal Recycling System Prior to Hijacking

Prior to establishing the complex infrastructure for intercellular mitochondrial hijacking described in the subsequent section, persister cells must weather the immediate metabolic shock of therapeutic intervention through an intrinsic survival mechanism called autophagy 84. Functioning as a critical internal recycling system, macroautophagy is rapidly induced in persister cells via the AMPK-mTOR signaling axis in response to nutrient deprivation and genotoxic stress 85. This catabolic process degrades non-essential proteins and damaged organelles into amino acids and fatty acids. These nutrients are subsequently funneled into the TCA cycle to maintain bioenergetic homeostasis during the dormant state 86. Furthermore, the interplay between autophagy and mitochondrial dynamics is pivotal. While persister cells generally favor mitochondrial hyperfusion to maximize efficiency and evade degradation, a selective form of autophagy known as mitophagy acts as a crucial quality control checkpoint 87. It specifically targets and prunes dysfunctional, ROS-generating mitochondria that fail to fuse, thereby preventing oxidative damage from triggering apoptosis. This obligatory reliance on autophagic flux for survival explains the rationale behind repurposing autophagy inhibitors, such as chloroquine or hydroxychloroquine, to sensitize persister cells to chemotherapy and targeted agents 88. By blocking this internal supply line, therapeutic strategies can force persister cells into metabolic collapse before they can successfully parasitize the microenvironment.

## 4. Metabolic Crosstalk in the Tumor Microenvironment: How Tumor Metabolism Suppresses Anti-Tumor Immunity

The metabolic reprogramming of cancer cells does not occur in isolation. It profoundly shapes the chemical composition of the tumor microenvironment, creating a hostile metabolic milieu that suppresses anti-tumor immunity. Effective immune surveillance, particularly by Cytotoxic T Lymphocytes, is energetically expensive and demands specific nutrients to sustain effector functions such as cytokine production and proliferation 89. However, the aggressive metabolic demands of the tumor driven by the Warburg and Reverse Warburg effects create a state of metabolic competition or nutrient deprivation. This competition acts as a non-immunological checkpoint. It forces T cells into states of anergy or exhaustion and facilitates immune escape 90.

### 4.1 Glucose Competition: The Tug-of-War for Energy

Effector T cells and tumor cells share a remarkably similar metabolic program. Upon activation, T cells must switch from oxidative phosphorylation to aerobic glycolysis to support rapid clonal expansion and the synthesis of effector molecules like Interferon-gamma and Granzyme B 91. This shared reliance on glycolysis places T cells in direct competition with highly glycolytic tumor cells for limited glucose resources. The consequence of this competition is a profound dampening of T cell signaling. Specifically, the depletion of glucose in the tumor microenvironment inhibits mTORC1 activity in T cells, which is the master regulator of glycolytic enzymes such as HK2 and effector function 92. When tumor cells consume the bulk of available glucose, T cells are metabolically crippled. This leads to reduced proliferation and a phenotype resembling exhaustion 93, 94. Furthermore, recent studies highlight a vicious cycle involving immune checkpoints. PD-L1 signaling on tumor cells has been shown to enhance tumor glycolysis via the Akt/mTOR pathway, actively starving T cells 95. Conversely, PD-1 ligation on T cells inhibits their glycolytic and amino acid metabolism, further reinforcing this suppressive state 96.

### 4.2 Lactate as an Immunosuppressive Signaling Molecule

**Figure 3:**
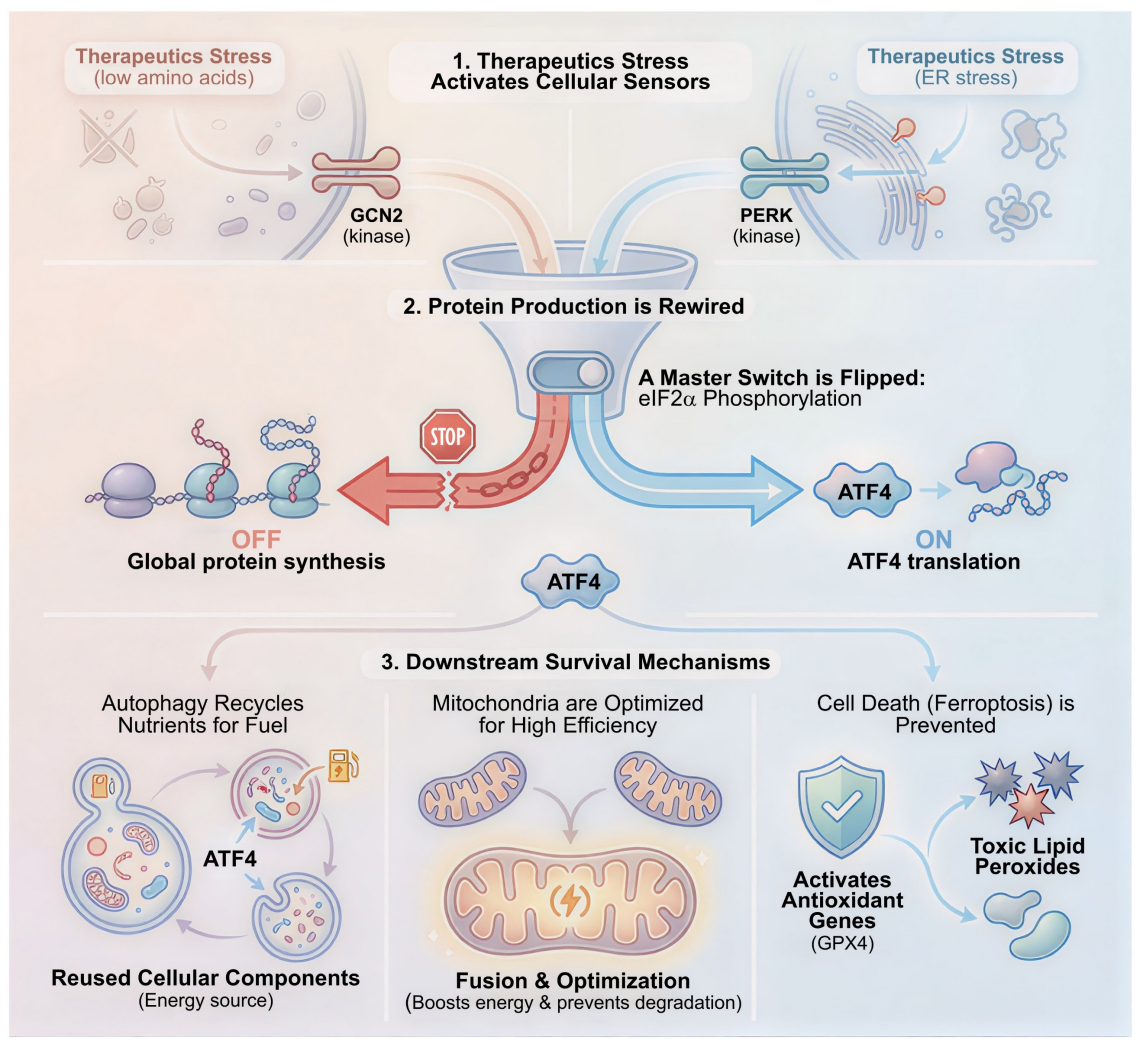
** The immunometabolic barrier: Metabolic competition and chemical suppression of anti-tumor immunity.** The figure illustrates the two-pronged metabolic assault on the immune microenvironment. Glucose Competition ("The Energy Heist"): High glycolytic rates in tumor cells deplete the TME of glucose, creating a nutrient desert. This deprivation starves infiltrating T cells, dampening mTORC1 signaling required for effector function and driving them towards an exhausted, anergic phenotype. The Lactate Barrier ("The Acid Attack"): Tumor-derived lactate accumulates in the extracellular space, creating an acidic milieu. This environment directly inhibits the cytotoxicity and proliferation of CD8+ T cells and NK cells while conversely metabolically supporting and activating immunosuppressive Regulatory T cells (Tregs) and M2 macrophages.

Beyond nutrient deprivation, the tumor microenvironment is often saturated with lactate produced by cancer-associated fibroblasts via the Reverse Warburg effect or by hypoxic tumor cells. While this lactate fuels oxidative tumor cells, it functions as a potent immunosuppressant for T cells. Mechanistically, high extracellular lactate concentrations disrupt the gradient required for T cells to export their own metabolic waste via MCT1. This blockage leads to the accumulation of intracellular lactate and protons, causing cytosolic acidification 97. This acidic environment directly inhibits the proliferation of CD8+ T cells and Natural Killer cells by suppressing the NFAT and p38/JNK signaling pathways. Thereby, it prevents the transcription of key cytokine genes like IFNG and IL2 98. Crucially, this environment creates a selective advantage for Regulatory T cells. Unlike effector T cells, Regulatory T cells are oxidatively poised and thrive in lactate-rich environments. The transcription factor FOXP3 reprograms Regulatory T cell metabolism to resist lactate toxicity and even utilize it for oxidative phosphorylation, thus tilting the immune balance towards tolerance 99 (**Figure 3**).

### 4.3 Therapy-Induced Nanotube Formation: The Stress Response

Metabolic reprogramming in DTPs is often triggered by the very therapies intended to eradicate them. While it is known that chemotherapy and radiotherapy induce DNA damage and mitochondrial dysfunction, a pivotal study in *Cancer Cell* recently demonstrated that these stressors also trigger a structural adaptation: the formation of Tunneling Nanotubes 100, 101. Tunneling Nanotubes are delicate, actin-based membranous channels that facilitate direct intercellular communication over long distances. Under the energy crisis induced by metabolic inhibitors or cytotoxic agents, tumor cells upregulate the machinery for nanotube formation as a survival reflex. This often involves proteins such as M-Sec, RalA, and Connexin-43. This structural extension acts as a desperate SOS signal. It allows compromised tumor cells to physically connect with healthy surrounding stromal fibroblasts or infiltrating immune cells. Crucially, this finding implies that the physical infrastructure for metabolic hijacking is not always pre-existing but is frequently constructed by the tumor in direct response to clinical intervention, creating a therapy-induced resistance network 102.

### 4.4 Intercellular Mitochondrial Hijacking

**Figure 4:**
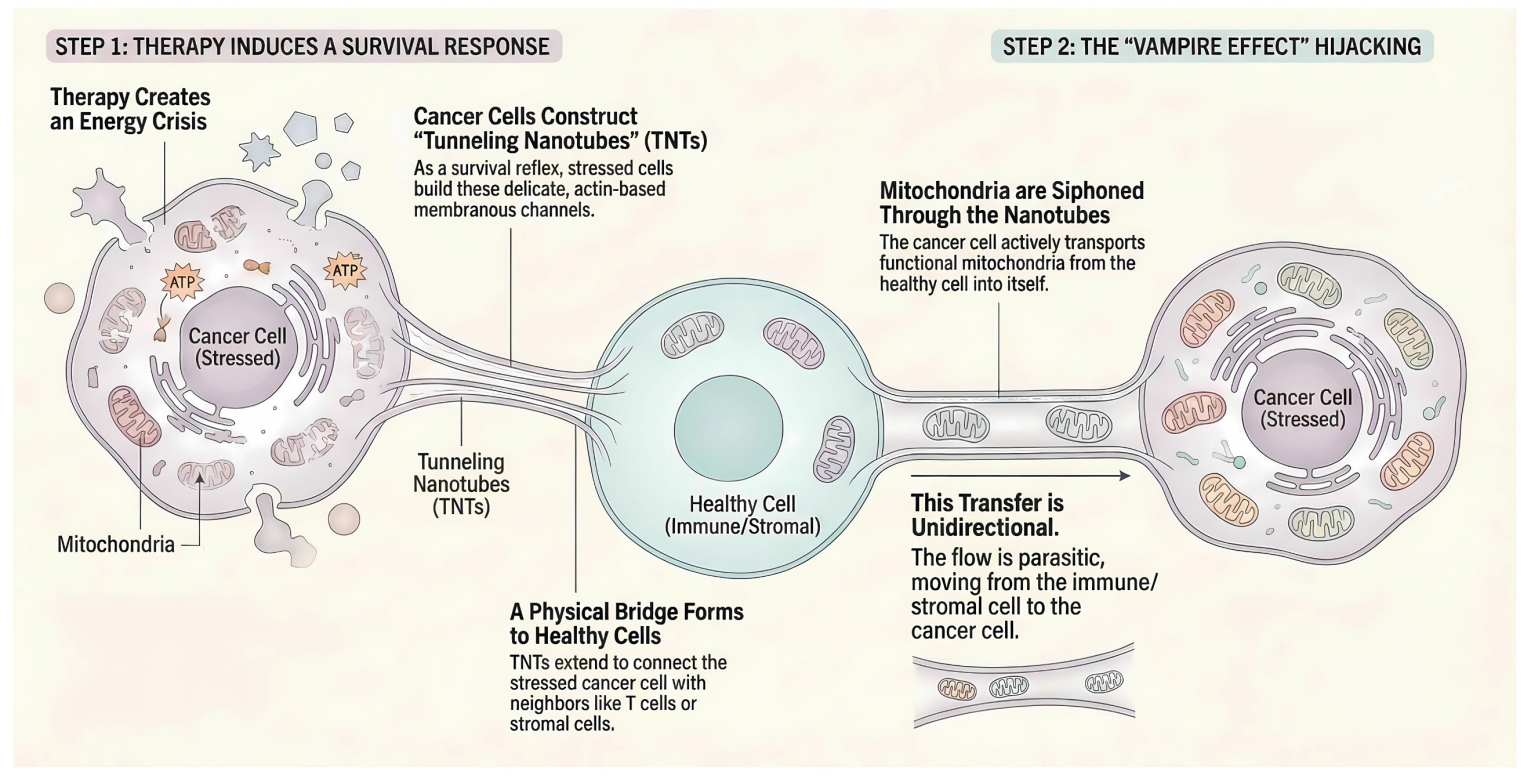
** Intercellular mitochondrial hijacking via tunneling nanotubes.** The figure illustrates the parasitic survival mechanism of DTPs under therapeutic stress. Therapy-Induced SOS: Chemotherapy or targeted agents create an acute energy crisis in cancer cells. As a survival reflex, these stressed cells upregulate actin polymerization to construct delicate membranous channels known as TNTs that physically connect with neighboring healthy cells (immune or stromal). Organelle Parasitism: Acting as metabolic parasites, DTPs utilize these conduits to unidirectionally siphon functional mitochondria from donor cells. This hijacking rapidly restores the bioenergetic machinery of the DTPs, enabling them to evade apoptosis and persist, while simultaneously depleting the metabolic fitness of the host microenvironment.

Once these physical conduits are established, DTPs exhibit a behavior best described as organelle parasitism. This fundamentally breaks the biological dogma that mitochondria are strictly inherited intracellularly. DTPs actively hijack functional mitochondria from neighboring cells to restore their own bioenergetic capacity. This process spans across different cell types in the microenvironment. In a landmark discovery, Saha et al. revealed that cancer cells utilize tunneling nanotubes to siphon mitochondria from infiltrating CD8^+^ T cells 103. This transfer is predominantly unidirectional, moving from the immune cell to the cancer cell. This allows DTPs to rapidly restore their oxidative phosphorylation capabilities and bypass the mitochondrial damage caused by initial therapy. Crucially, this predatory behavior extends beyond the immune landscape to the structural niche. As elucidated in a recent *Nature Cancer* study, glioblastoma cells form robust Tumor Microtubes to integrate into a multicellular network with neurons and astrocytes. They scavenge mitochondria from the brain stroma to repair radiation-induced damage 104, 105. Similarly, in hematological malignancies like multiple myeloma, CD38 signaling drives the uptake of mitochondria from bone marrow stromal cells via tunneling nanotubes, directly fueling the tumor's bioenergetic needs 106. Collectively, this acquisition of exogenous mitochondria effectively resets the metabolic state of DTPs. It acts as an external backup system that ensures survival even when the tumor's own metabolic machinery is compromised. Collectively, this acquisition of exogenous mitochondria effectively resets the metabolic state of DTPs, acting as an external backup system that ensures survival even when the tumor's own metabolic machinery is compromised (**Figure 4**).

### 4.5 Metabolic Paralysis of the Immune System

The consequence of this hijacking is a double whammy for the host. While the tumor gains a metabolic boost, the antitumor immune response suffers a catastrophic collapse. The transfer of mitochondria leads to a state of metabolic exhaustion in the donor T cells that is distinct from, yet additive to, traditional checkpoint-mediated exhaustion. T cells rely heavily on mitochondrial flexibility to switch between glycolysis for effector function and oxidative phosphorylation for memory formation 107. When their mitochondria are physically extracted by DTPs, T cells experience a sharp decline in respiratory capacity and reactive oxygen species production. This renders them unable to synthesize cytokines or proliferate upon antigen stimulation 108. Effectively, the tumor removes the T cell's engine, making them unresponsive to immune checkpoint blockade such as anti-PD-1 therapy. Furthermore, the reinvigorated metabolism of DTPs exacerbates the hostility of the tumor microenvironment. The strengthened oxidative phosphorylation and subsequent Reverse Warburg activity lead to the accumulation of lactate and protons in the extracellular space. This acidification suppresses the cytotoxic activity of NK and T cells while promoting the polarization of macrophages towards an immunosuppressive M2 phenotype 109. Thus, the metabolic shapeshifting of DTPs serves a dual purpose. It acts as an intrinsic shield against targeted therapy and an extrinsic weapon that disarms the immune system.

### 4.6 Lipid Metabolism and Myeloid Skewing

Lipid metabolism plays a pivotal role in shaping the innate immune compartment, specifically causing the skewing of Tumor-Associated Macrophages and Myeloid-Derived Suppressor Cells. The accumulation of lipids in the tumor microenvironment, often derived from aberrant lipogenesis or increased fatty acid uptake via CD36, drives macrophages towards an anti-inflammatory M2 phenotype 110. These M2 macrophages rely heavily on Fatty Acid Oxidation and oxidative phosphorylation, a metabolic state driven by PGC-1β and STAT6 signaling 111, 112. Functionally, these lipid-loaded macrophages secrete immunosuppressive cytokines such as IL-10 and TGFβ, along with high levels of Arginase-1. Arginase-1 depletes arginine from the microenvironment, an amino acid essential for T cell receptor signaling complex stability, specifically the zeta-chain 113. This creates a dangerous synergy. DTP cells and M2 macrophages both rely on Fatty Acid Oxidation, forming a lipid-dependent and immunosuppressive niche that protects residual disease from immune clearance 114.

### 4.7 Therapeutic Implications: Metabolic Checkpoint Inhibitors

Understanding these mechanisms suggests that metabolic interventions could serve as critical partners to revitalize immunotherapy. Therapeutic strategies are now focusing on Metabolic Checkpoint Inhibitors. For instance, buffering the acidic tumor pH using bicarbonate or proton pump inhibitors has been shown to restore T cell function and improve the efficacy of anti-PD-1 and anti-CTLA-4 antibodies in preclinical models 115. Similarly, MCT1/4 inhibitors like AZD3965 serve a dual purpose. They starve oxidative tumor cells while potentially restoring the lactate gradient for T cells, effectively un-choking their metabolism 116. Furthermore, targeting lipid uptake via CD36 inhibitors or blocking Fatty Acid Oxidation, for example with Etomoxir, can reprogram immunosuppressive M2 macrophages back to an anti-tumor M1 phenotype. Thereby, breaking the immunosuppressive barrier and resensitizing the tumor to immune attack 117.

## 5. Therapeutic Opportunities: Breaking the Symbiosis and Targeting Plasticity

Therapeutic strategies targeting cancer metabolism have evolved from general anti-metabolites to precise, mechanism-based inhibitors designed to exploit specific metabolic vulnerabilities. Given the metabolic plasticity described above, monotherapy is rarely curative. However, targeting metabolic hubs offers a potent strategy to sensitize tumors to standard-of-care agents and eradicate DTP cells 118. Here, we discuss specific small molecules currently under clinical investigation that target the key nodes of metabolic flexibility. A summary of these metabolic inhibitors and their current clinical development status is provided in **Table 1**.

### 5.1 Disrupting the Lactate Shuttle: MCT1 Inhibitors

Targeting the Reverse Warburg phenotype requires blocking the uptake of stromal-derived lactate by oxidative tumor cells. The most advanced agent in this class is AZD3965, a first-in-class and selective oral inhibitor of Monocarboxylate Transporter 1. Preclinical data demonstrated that AZD3965 effectively inhibits lactate transport, leading to intracellular acidification and cessation of TCA cycle activity in lactate-dependent tumors 116. In a Phase I clinical trial (NCT01791595), AZD3965 showed manageable toxicity and evidence of target engagement in advanced solid tumors 119, 120. However, a critical mechanism of resistance identified is the compensatory upregulation of MCT4, which can export lactate even when MCT1 is blocked 25. Consequently, clinical strategies are now shifting towards patient stratification by selecting tumors with high MCT1 and low MCT4 expression or developing dual MCT1/4 inhibitors to fully abrogate the lactate shuttle 24.

### 5.2 Starving the TCA Cycle: Glutaminase and Dual-Targeting

When glucose utilization is blocked, for example by the Warburg effect or therapy, cancer cells frequently exhibit glutamine addiction to refuel the TCA cycle. Telaglenastat (CB-839) is a selective, oral inhibitor of glutaminase that showed promising preclinical activity by depriving tumors of a major anaplerotic input 121. However, early clinical experience cautions against overinterpreting preclinical single-agent efficacy. The Phase II CANTATA trial (CB-839 + cabozantinib vs placebo + cabozantinib in advanced RCC) did not meet its primary endpoint, although exploratory subgroup analyses hinted at benefit in patients with specific metabolic signatures 122, 123. Mechanistically, several factors likely constrained clinical impact: (i) metabolic redundancy — tumors can employ alternative anaplerotic routes (increased uptake or synthesis of asparagine, upregulation of transaminases, or increased macropinocytosis) to refill the TCA cycle when glutaminase is inhibited; (ii) intratumoral heterogeneity and patient selection — bulk genomic or histologic selection does not reliably identify tumors that are truly glutamine-addicted in vivo, and standard imaging (FDG-PET) poorly captures non-glucose fuel usage; and (iii) pharmacodynamic limitations and tolerability windows that constrain the exposures achievable in patients. Together, these limitations argue that glutaminase inhibition will be most effective when (a) patients are stratified by dynamic metabolic biomarkers (for example, ¹⁸F-glutamine PET or functional metabolomic assays), (b) glutaminase inhibitors are combined with agents that block compensatory anaplerotic pathways or that generate synthetic lethal dependencies, and (c) dosing is timed to eliminate persister cells during a co-extinction window rather than used as late-line monotherapy. These lessons directly support our advocacy for metabolic priming and combination/siege strategies rather than expectation of single-agent cures.

### 5.3 Disrupting the Hijacking Infrastructure

Given that DTPs rely on the physical acquisition of exogenous mitochondria to survive therapeutic stress, a novel therapeutic frontier lies in severing these intercellular supply lines to quarantine the tumor cells. Since the machinery of mitochondrial trafficking involves specific surface markers and cytoskeletal structures, it presents unique druggable targets. Clinically, the most immediate strategy involves repurposing agents that block the molecular engines of transfer. For instance, the FDA-approved anti-CD38 antibody, Daratumumab, has been shown to inhibit the formation of tunneling nanotubes and mitochondrial uptake in hematological malignancies, effectively starving the tumor of stromal support 106. Beyond surface markers, targeting the structural integrity of these conduits offers another avenue. While global cytoskeleton inhibition is toxic, more precise approaches focus on the gap junction protein Connexin-43, which facilitates metabolic coupling within these tubes. Agents like tonabersat or specific mimetic peptides can functionally isolate persister cells from the astrocytic or stromal network, preventing the rescue effect of hijacked mitochondria 103. Furthermore, upstream inhibition of the mTOR pathway is being explored as a strategy to suppress stress-induced adaptive programs, including intercellular communication mechanisms such as nanotube formation. However, given the essential role of mTOR signaling in T-cell activation, differentiation, and effector function, such approaches will require careful evaluation to avoid unintended immunosuppressive effects in the tumor microenvironment 47.

### 5.4 Revitalizing Immunometabolism

To overcome the metabolic paralysis of T cells described previously, therapeutic strategies must go beyond targeting the tumor cells to actively restoring the metabolic fitness of the immune infiltrate. This concept gives rise to the class of Metabolic Checkpoint Inhibitors designed to reverse the hostile microenvironmental conditions. A primary focus is buffering the acidic tumor pH driven by lactate accumulation. Systemic buffering agents, such as oral bicarbonate or proton pump inhibitors, have been shown to neutralize the extracellular space, allowing T cells to export their own metabolic waste and restoring their cytokine production capabilities 97. Concurrently, targeting the adenosine axis is critical to lifting metabolic suppression. The breakdown of ATP in the hypoxic tumor microenvironment leads to the accumulation of adenosine, which binds to A2A receptors on T cells to inhibit activation. Novel A2A receptor antagonists, such as Ciforadenant, prevent this signal transduction and are currently being evaluated in combination with anti-PD-1 therapies 128. By combining pH buffering with adenosine blockade, these strategies aim to un-choke the T cell metabolism, enabling them to mount an effective attack against the weakened persister cells.

### 5.5 Direct Inhibition of OXPHOS: Targeting the Persisters

Inhibitors of the mitochondrial electron transport chain have clear scientific rationale for targeting OXPHOS-addicted persisters, but their clinical translation has thus far exposed important limitations. IACS-010759, a potent complex I inhibitor, demonstrated strong preclinical efficacy in models dependent on oxidative metabolism 60, yet early clinical development revealed a narrow therapeutic index characterized by dose-limiting toxicities including elevated blood lactate and neurotoxicity that constrained tolerable exposures in patients 131, 132. Mechanistically, systemic inhibition of a core bioenergetic pathway risks on-target effects in high-metabolic-demand normal tissues (brain, heart, skeletal muscle), and the plasma exposures required for robust complex I blockade in vitro are frequently close to or above tolerability thresholds in humans. In addition, tumors can counter ETC blockade via compensatory glycolytic reprogramming or selection of clones with alternate metabolic wiring, limiting single-agent durability. IM156 and other next-generation OXPHOS modulators aim to widen this therapeutic window, but early human data indicate modest monotherapy activity in unselected populations 133-135,136.

Taken together, clinical experience with IACS-010759 argues for four pragmatic design principles: (1) careful patient selection using functional metabolic biomarkers to identify truly OXPHOS-dependent tumors; (2) temporal sequencing/pulsed dosing (extinction pulses) to eliminate persisters when tumor burden and systemic exposure risk are most favorable; (3) tumor-targeted delivery (prodrugs, nanocarriers, or tumor-activated triggers) to minimize CNS and cardiac exposure; and (4) rational combinations that prevent compensatory glycolysis (for example, transient inhibition of glycolytic flux in the co-extinction window or simultaneous targeting of biosynthetic escape routes). We have expanded Section 6.4 (Managing Toxicity and Therapeutic Windows) to incorporate these translational caveats and design recommendations.

### 5.6 Exploiting Ferroptosis Vulnerabilities

Given that DTPs accumulate lipids and are dependent on GPX4 for survival, inducing ferroptosis is an emerging therapeutic frontier. While specific GPX4 inhibitors like RSL3 are largely preclinical tools due to pharmacokinetics, existing drugs are being repurposed 55. Sorafenib, a multi-kinase inhibitor approved for liver cancer, has been found to induce ferroptosis by inhibiting System xc-, the cystine/glutamate antiporter required for glutathione synthesis 139. Newer generation agents and combination strategies aimed at amplifying lipid peroxidation in metabolically plastic tumors are currently a major focus of drug discovery efforts 73, 140.

### 5.7 Metabolic Plasticity as a Major Barrier to Durable Metabolic Inhibition

**Figure 5:**
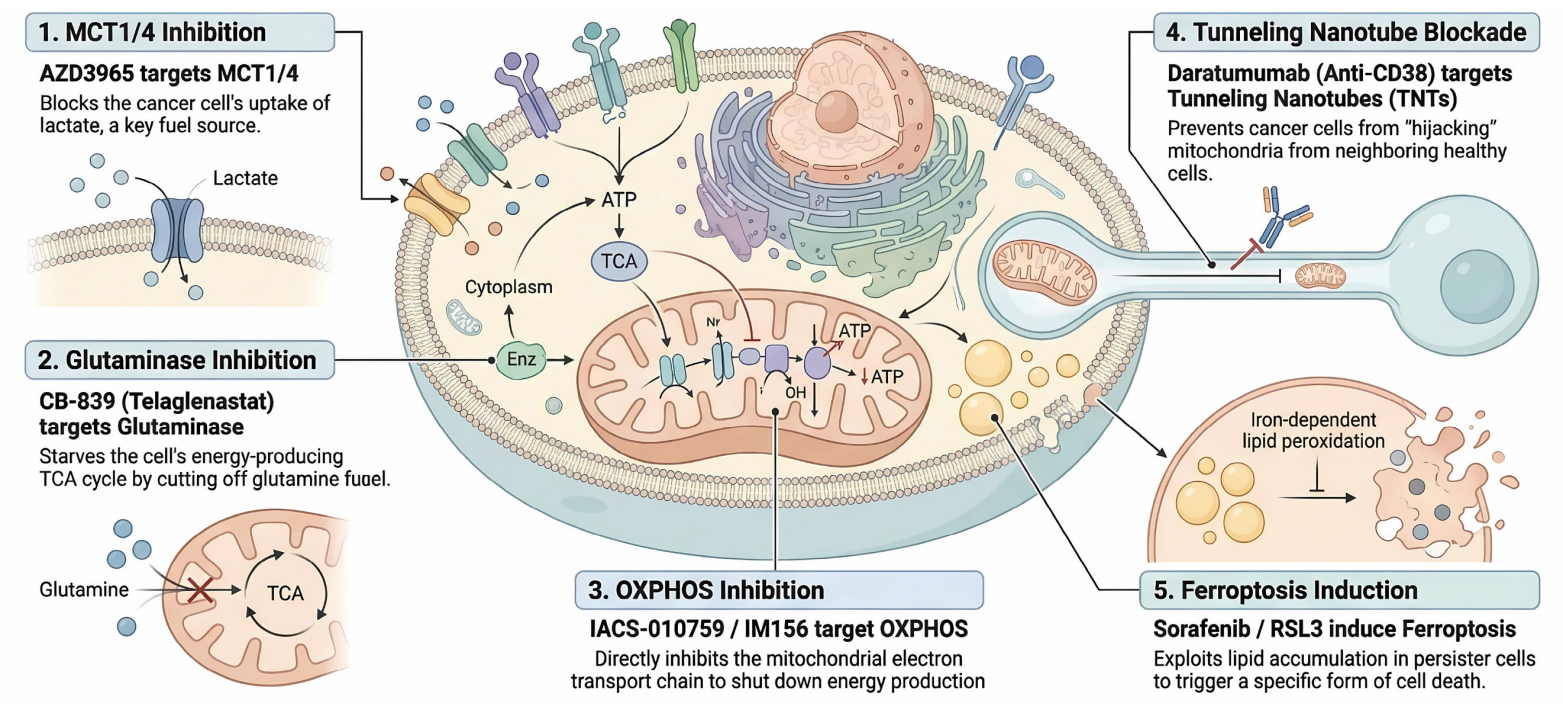
** Therapeutic strategies targeting metabolic vulnerabilities and plasticity.** The schematic summarizes key pharmacological interventions designed to exploit the specific bioenergetic dependencies of DTPs. (1) Disrupting the Lactate Shuttle: MCT1/4 inhibitors (AZD3965) block the uptake of stromal-derived lactate, depriving oxidative tumor cells of a critical fuel source. (2) Starving the TCA Cycle: Glutaminase inhibitors (CB-839) prevent the conversion of glutamine to glutamate, thereby cutting off anaplerotic fueling of the TCA cycle. (3) Direct OXPHOS Inhibition: Small molecule inhibitors of the electron transport chain (IACS-010759, IM156) directly suppress mitochondrial respiration, targeting the "mitochondria-addicted" phenotype of DTPs. (4) Blockade of Mitochondrial Hijacking: Agents targeting structural or signaling components of TNTs, such as the anti-CD38 antibody Daratumumab, sever the physical connections required for scavenging exogenous mitochondria. (5) Ferroptosis Induction: Exploiting the high lipid dependency of DTPs, agents like Sorafenib or RSL3 promote the accumulation of lethal lipid peroxides, triggering ferroptotic cell death.

The clinical failures and limited single-agent efficacy observed with first-generation metabolic inhibitors underline a critical principle: metabolic redundancy and compensatory adaptation are central obstacles. For example, glutaminase blockade by CB-839 can be bypassed via upregulation of asparagine synthetase or alternative transaminase pathways that replenish TCA intermediates, allowing tumors to sustain mitochondrial anaplerosis 141-143. Similarly, lactate uptake blockade through MCT1 inhibition can be neutralized by compensatory MCT4 upregulation or metabolic switching toward fatty acid oxidation 154. These interconnected compensatory circuits, spanning intrinsic tumor metabolism and extrinsic stromal support, are schematically summarized in **Figure 5**. Importantly, clinical experiences (the CANTATA program for CB-839 and the dose-limiting toxicities encountered with IACS-010759) demonstrate that these escape mechanisms are not merely theoretical but materially constrain therapeutic windows in patients. Therefore, effective strategies must combine (i) dynamic metabolic patient selection, (ii) rational dual- or multi-node metabolic blockade to prevent immediate compensation, and (iii) temporal sequencing (metabolic priming/co-extinction) to eradicate persister reservoirs before genetic resistance emerges. These practical recommendations constitute the clinical corollary of the “metabolic siege” framework proposed in this review.

## 6. Future Perspectives and Conclusion

While the targeting of metabolic plasticity represents a scientifically rational frontier in oncology, the translation of these concepts into durable clinical benefits remains a formidable challenge. The limited success of first-generation metabolic inhibitors highlights a critical lesson that tumor metabolism is not a static target but a dynamic network capable of rapid rewiring. To realize the potential of metabolic therapy, future research and clinical trial design must pivot towards precision medicine and dynamic monitoring.

### 6.1 From Static to Dynamic Biomarkers

A major impediment to current trials is the lack of predictive biomarkers. Currently, patients are often selected based on histology or genetic mutations, which do not necessarily reflect metabolic phenotypes. The standard FDG-PET scan detects glucose-avid Warburg tumors but fails to visualize the lactate-dependent or lipid-reliant populations, such as reverse Warburg or DTP cells, that are responsible for resistance 146. The integration of novel PET tracers, such as 18F-Glutamine or 11C-Acetate for Fatty Acid Oxidation, is essential to stratify patients who will benefit from glutaminase or fatty acid oxidation inhibitors 147. Furthermore, the concept of a "Metabolomic biopsy," which involves analyzing circulating tumor DNA alongside circulating metabolites, could provide a real-time snapshot of the tumor's metabolic switch before radiographic progression occurs 148.

### 6.2 Systemic Metabolic Interventions: Diet as a Precision Adjuvant

Targeting intrinsic tumor metabolism without considering host systemic physiology often leads to therapeutic failure due to compensatory hormonal feedback loops. A paradigm-shifting example is the interaction between PI3K inhibitors and systemic glucose homeostasis. The inhibition of PI3K signaling in skeletal muscle blocks glucose uptake, leading to acute hyperglycemia. This triggers a compensatory surge in host insulin production. This insulin surge reactivates PI3K signaling in the tumor via insulin receptors, thereby abrogating the drug's efficacy 149. Recent studies demonstrate that a Ketogenic Diet, which suppresses systemic insulin levels, can break this feedback loop and significantly enhance the efficacy of PI3K inhibitors in drug-resistant tumors. Beyond insulin modulation, Fasting-Mimicking Diets or periodic fasting impose a systemic nutrient stress that exploits the concept of Differential Stress Resistance. While normal cells respond to nutrient deprivation by entering a protected maintenance mode by downregulating proliferation pathways like IGF-1 and mTOR, oncogene-driven cancer cells lack this metabolic flexibility. They remain committed to growth, rendering them vulnerable to oxidative stress and chemotherapy 150. However, a nuanced approach is required in the context of DTPs. Given that these cells are reliant on Fatty Acid Oxidation, high-fat ketogenic interventions could theoretically fuel their persistence if used in isolation. Therefore, the future of systemic intervention lies in Diet-Drug Synergy. Strategies such as combining ketogenic diets specifically with FAO inhibitors or energetic stress inducers can simultaneously cut off both glucose via diet and lipid utilization via drug 151. This leaves the metabolic shapeshifters with no escape route.

### 6.3 Timing and Sequencing: The Concept of "Metabolic Priming"

Most clinical trials administer metabolic inhibitors as a salvage therapy in late-stage and heavily pre-treated patients. However, the biology of DTPs suggests that metabolic plasticity is engaged immediately upon exposure to targeted therapy. We propose a paradigm shift towards Metabolic Priming or Co-extinction strategies. Instead of waiting for resistance to emerge, metabolic inhibitors such as OXPHOS or FAO inhibitors should be administered upfront or in a pulsatile manner alongside the primary kinase inhibitor. This approach aims to eliminate the reservoir of metabolically adaptable cells before they can establish a genetically resistant clone 134.

### 6.4 Managing Toxicity and Therapeutic Windows

Targeting mitochondria or glycolysis inherently risks toxicity in metabolically active healthy tissues, including the heart, brain, and skeletal muscle, as these tissues share fundamental bioenergetic dependencies with tumor cells. As a result, achieving sufficient target inhibition in cancer cells without compromising normal tissue function remains a central challenge for the clinical translation of metabolic therapies. An important clinical limitation of targeting mitochondrial metabolism is the risk of on-target toxicity in tissues with high energetic demand, particularly the central nervous system. Clinical experience with complex I inhibitors such as IACS-010759 has demonstrated dose-limiting neurotoxicity and systemic metabolic adverse events, underscoring the narrow therapeutic window of systemic OXPHOS inhibition 131, 132. These observations highlight that toxicity is not merely an off-target concern, but a predictable consequence of inhibiting core mitochondrial functions shared by normal tissues. To widen the therapeutic index of metabolic interventions, several mitigation strategies are being explored. These include tumor-activated prodrugs and nanocarrier-based delivery systems that exploit tumor-specific features such as acidic pH or elevated ROS levels 152, as well as functional metabolic patient selection, temporally restricted or pulsed dosing schedules, and tumor-selective delivery approaches. In parallel, dietary interventions, including ketogenic or fasting-mimicking diets, offer non-pharmacological means to modulate systemic nutrient availability, potentially sensitizing tumors to metabolic drugs while protecting normal tissues 153. Collectively, these principles are essential for translating OXPHOS-targeted therapies into clinically viable components of combination or extinction-based treatment regimens.

### 6.5 Concluding Remarks

**Figure 6:**
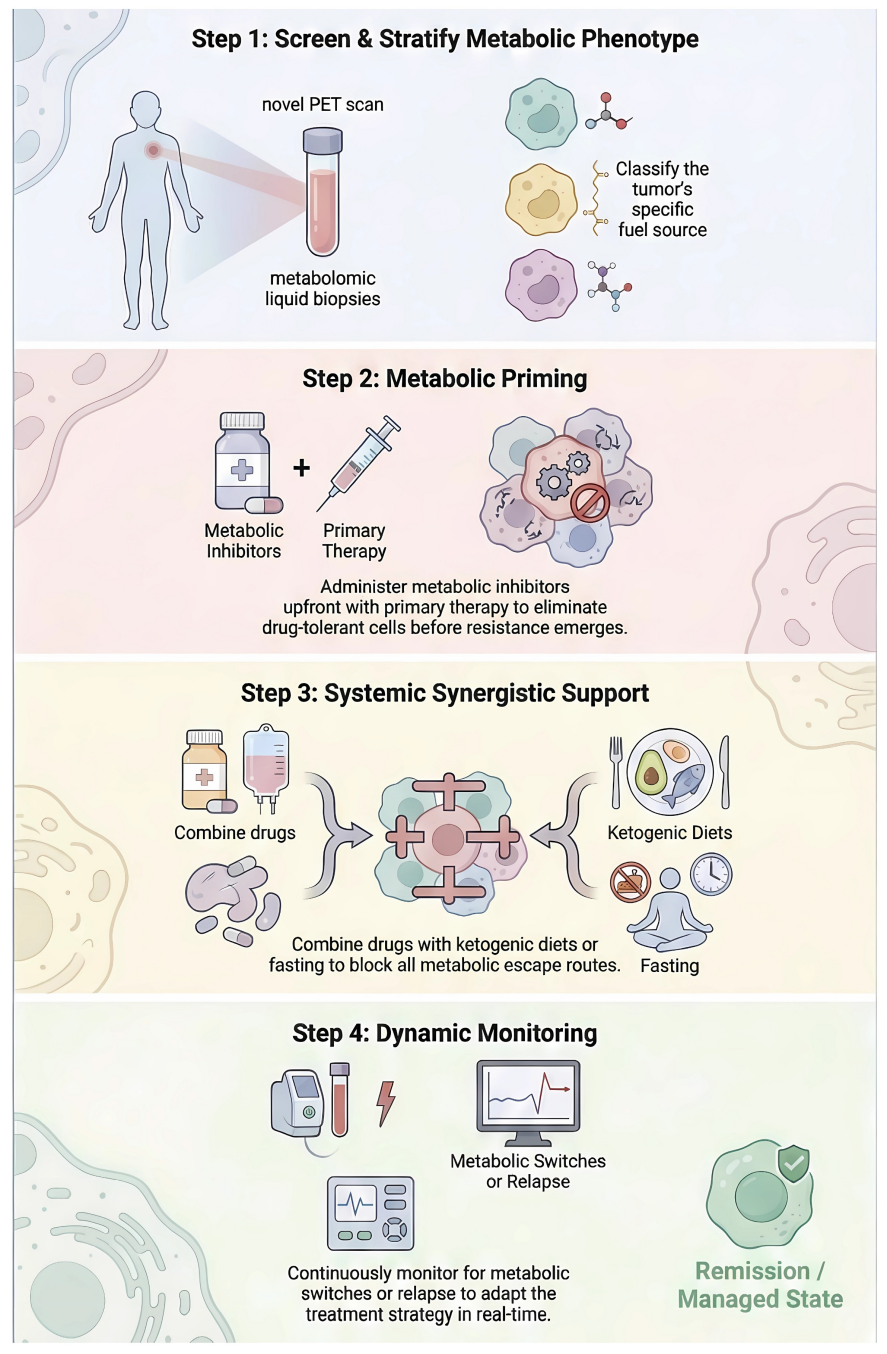
** A clinical roadmap for overcoming metabolic resistance and targeting plasticity.** The diagram outlines a four-step paradigm shift towards precision metabolic oncology to prevent DTP-driven relapse. (Step 1) Precision Metabolic Screening: Moving beyond static histology, this step integrates novel PET tracers and liquid biopsies to stratify tumors based on their specific bioenergetic dependencies (glucose-avid vs. lipid-reliant). (Step 2) Metabolic Priming: A therapeutic sequencing strategy where metabolic inhibitors are administered upfront or alongside primary kinase inhibitors. This "co-extinction" approach aims to eradicate the reservoir of metabolically plastic persister cells before they can evolve genetic resistance. (Step 3) Systemic Support: Implementation of "Diet-Drug Synergy," such as Ketogenic Diets or Fasting-Mimicking Diets, to manipulate systemic nutrient availability (lowering insulin), thereby cutting off compensatory metabolic escape routes. (Step 4) Dynamic Monitoring: Continuous assessment of metabolic switching via circulating biomarkers allows for real-time therapeutic adaptation ("Whac-A-Mole" strategy) to preemptively manage relapse.

The journey from the Warburg Effect to the Reverse Warburg Effect and the discovery of DTPs has revealed the sophisticated metabolic intellect of cancer cells. We now understand that resistance is not merely a genetic inevitability but a metabolic choice. By dissecting the signaling nodes that govern this plasticity, from stromal-epithelial lactate shuttles to mitochondrial remodeling, we uncover a new dimension of therapeutic targets. The next generation of cancer therapy will likely not rely on a single “magic bullet,” but instead on a strategic metabolic siege that dismantles the tumor's capacity to adapt, evolve, and survive (**Figure 6**). Future studies should prioritize temporally resolved metabolic profiling of persister populations, develop functional biomarkers to guide metabolic patient stratification, and test multi-layer therapeutic designs that simultaneously constrain tumor-intrinsic, stromal, and systemic metabolic inputs.

## Figures and Tables

**Table 1:** Selected Clinical Trials of Metabolic Inhibitors Targeting Plasticity

Drug Target	Drug Name	Mechanism Of Action	Clinical Status (Phase)	Combination Strategy
Mct1	AZD3965	Inhibits lactate uptake (Reverse Warburg)	Phase I (Completed)	w/ Chemotherapy or TKIs
Glutaminase	Telaglenastat (CB-839)	Blocks Glutamine to Glutamate	Phase II (CANTATA)	w/ Cabozantinib / Immunotherapy
Complex I	IACS-010759	Inhibits OXPHOS / ETC	Phase I (Discontinued due to tox)	Monotherapy / w/ Azacitidine
Complex I	IM156	Inhibits OXPHOS (Biguanide)	Phase I	w/ Standard of Care
Tca Cycle	Devimistat (CPI-613)	Disrupts mitochondrial energy flux	Phase III	w/ Chemotherapy (AML)
Cd38	Daratumumab	Inhibits mitochondrial transfer (Hijacking)	Phase III	w/ Proteasome Inhibitors
Adenosine A2a	Ciforadenant	Blocks adenosine immunosuppression	Phase I/II	w/ Anti-PD-1 (Pembrolizumab)
